# COVID-19 Outbreaks and Mortality Among Public Transportation Workers — California, January 2020–May 2022

**DOI:** 10.15585/mmwr.mm7133a4

**Published:** 2022-08-19

**Authors:** Amy Heinzerling, Ximena P. Vergara, Elisabeth Gebreegziabher, John Beckman, Jessie Wong, Alyssa Nguyen, Sana Khan, Matt Frederick, David Bui, Elena Chan, Kathryn Gibb, Andrea Rodriguez, Seema Jain, Kristin J. Cummings

**Affiliations:** ^1^California Department of Public Health; ^2^Heluna Health, City of Industry, California; ^3^Public Health Institute, Oakland, California.

Work-related factors can contribute to risk for exposure to and infection with SARS-CoV-2, the virus that causes COVID-19, and subsequent COVID-19–attributable outcomes, including death. Comparing COVID-19 metrics across industries can help identify workers at highest risk. Elevated COVID-19 mortality rates have been reported among all transportation workers, as well as specifically in public transportation industries (*1*–*3*). The California Department of Public Health (CDPH) calculated public transportation industry–specific COVID-19 outbreak incidence during January 2020–May 2022 and analyzed all laboratory-confirmed COVID-19 deaths among working-age adults in California to calculate public transportation industry–specific mortality rates during the same period. Overall, 340 confirmed COVID-19 outbreaks, 5,641 outbreak-associated cases, and 537 COVID-19–associated deaths were identified among California public transportation industries. Outbreak incidence was 5.2 times as high (129.1 outbreaks per 1,000 establishments) in the bus and urban transit industry and 3.6 times as high in the air transportation industry (87.7) as in all California industries combined (24.7). Mortality rates were 2.1 times as high (237.4 deaths per 100,000 workers) in transportation support services and 1.8 times as high (211.5) in the bus and urban transit industry as in all industries combined (114.4). Workers in public transportation industries are at higher risk for COVID-19 workplace outbreaks and mortality than the general worker population in California and should be prioritized for COVID-19 prevention strategies, including vaccination and enhanced workplace protection measures.

This report assessed confirmed COVID-19 outbreaks in California workplaces that began during January 1, 2020–May 26, 2022, and were reported to CDPH as of June 27, 2022. Confirmed COVID-19 outbreaks were defined as the occurrence of three or more probable or confirmed COVID-19 cases within a 14-day period among persons who are epidemiologically linked in the setting, are from different households, and are not identified as close contacts of one another in any other case investigation (*4*). Since January 1, 2021, California employers have been required to report workplace clusters of three or more COVID-19 cases within 14 days to their local health department (LHD); previously, outbreak reporting requirements varied by setting and jurisdiction. LHDs report confirmed COVID-19 outbreaks and the number of outbreak-associated cases, which might include workers and nonworkers, to CDPH.

Separately, deaths among persons with laboratory-confirmed* COVID-19 were ascertained from California’s COVID-19 case registry, using LHD determinations to identify COVID-19 decedents.^†^ Case registry records were probabilistically matched to state death certificate data, which include information about decedent industry and occupation. COVID-19 decedents with date of death during January 1, 2020–May 26, 2022, were analyzed; analysis was restricted to working adults aged 18–64 years.

Standard 2012 U.S. Census Bureau industry codes were manually assigned to outbreaks using employer information and were assigned to death certificate free text for “usual industry” using an automated coding system (*5*). The numbers of outbreaks, outbreak-associated cases, and COVID-19–associated deaths were calculated for public transportation industries overall and for the five included individual industries^§^: air transportation, rail transportation, bus service and urban transit, taxi and limousine service (including shared ride services), and transportation support services (e.g., transportation maintenance services and airport cargo or terminal services).

For industries with 10 or more outbreaks during the study period, industry-specific outbreak incidence, defined as number of outbreaks per 1,000 business establishments, was calculated, using data on numbers of establishments from the California Employment Development Department in 2020 as denominators.^¶^ Monthly employment data from the U.S. Census Bureau’s 2020–2022 Current Population Survey were used as denominators to calculate industry-specific annual, cumulative, and age-standardized mortality rates. Outbreak incidence and mortality rates in public transportation industries were compared to overall rates for all California industries combined. Analyses were performed using SAS software (version 9.4; SAS Institute). The California Health and Human Services Agency’s Committee for the Protection of Human Subjects determined that this project constituted public health practice, not research, and therefore did not require further human subjects review.**

A total of 340 COVID-19 outbreaks, 5,641 outbreak-associated cases, and 537 COVID-19–associated deaths occurred in public transportation industries in California (Table 1) (Table 2). The largest number of outbreaks (194; 57.1%) occurred in bus and urban transit workplaces, the largest number of outbreak-associated cases occurred in air transportation (2,411; 42.7%), and the largest number of deaths (270; 50.3%) occurred among workers in transportation support services.

**TABLE 1:** COVID-19 outbreaks and outbreak incidence* in public transportation industries — California, January 2020–May 2022

Industry	No. of outbreaks				No. (%) of outbreak-associated cases^†^	No. of establishments*	Annual outbreak incidence*		Cumulative outbreak incidence*
	2020	2021	2022	Total (%)^†^			2020	2021	
Air transportation	16	31	6	**53 (15.6)**	2,411 (42.7)	604	26.5	51.3	87.7
Rail transportation^§^	0	4	0	**4 (1.2)**	48 (0.9)	9	NA	NA	NA
Bus service and urban transit	73	97	24	**194 (57.1)**	2,129 (37.7)	1,502	48.6	64.5	129.1
Taxi and limousine service^§^	0	0	0	**0 (—)**	0 (—)	770	NA	NA	NA
Transportation support services	24	58	7	**89 (26.2)**	1,053 (18.7)	6,755	3.6	8.6	13.2
**All public transportation industries**	**113**	**190**	**37**	**340 (100)**	**5,641 (100)**	**9,772**	**11.7**	**19.7**	**35.3**
**All industries**	**13,571**	**16,572**	**10,078**	**40,221**	**495,427**	**1,629,893**	**8.3**	**10.2**	**24.7**

**Abbreviation:** NA = not applicable.

* Outbreak incidence was calculated as number of outbreaks per 1,000 establishments; an annual rate was not calculated for 2022 because of incomplete data. Cumulative outbreak incidence was calculated as total number of outbreaks during the study period per 1,000 establishments. Establishment numbers reflect 2020 data because complete data for 2021–2022 were not available; 2020 establishments were extrapolated for 2021 and cumulative incidence calculations.

^†^ Percentages were calculated as percentages of outbreaks or outbreak-associated cases among all public transportation industries.

^§^ Outbreak incidence was not calculated for industries with small numbers (<10) of reported outbreaks.

**TABLE 2:** COVID-19 deaths and mortality rates* among workers in public transportation industries — California, January 2020–May 2022

Industry	No. of COVID-19 deaths				Total no. of workers (x 1,000)	Annual mortality rate*		Cumulative mortality rate*
	2020	2021	2022	Total (%)^†^		2020	2021	
Air transportation	18	34	5	**57 (10.6)**	62.5	31.2	68.0	91.3
Rail transportation	10	23	4	**37 (6.9)**	15.3	51.5	230.1	241.8
Bus service and urban transit	33	63	10	**106 (19.7)**	50.1	76.6	114.3	211.5
Taxi and limousine service	26	35	6	**67 (12.5)**	66.6	31.4	58.3	100.6
Transportation support services	92	157	21	**270 (50.3)**	113.8	99.5	132.2	237.4
**All public transportation industries**	**179**	**312**	**46**	**537 (100)**	**308.3**	**60.6**	**106.1**	**174.2**
**All industries**	**6,330**	**11,567**	**2,455**	**20,442**	**17,875.3**	**35.6**	**65.2**	**114.4**

* Mortality rates were calculated as number of deaths per 100,000 workers and were not calculated for 2022 because of incomplete annual data. Reported rates are crude mortality rates. Cumulative mortality rate was calculated as total number of deaths during the study period per 100,000 workers, using average employment during the study period.

^†^ Percentages were calculated as percentages of deaths among all public transportation industries.

During January 1, 2020–May 26, 2022, the cumulative outbreak incidence for all public transportation industries (35.3 outbreaks per 1,000 establishments) was 1.4 times as high as that for all industries (24.7) (Table 1). Among individual public transportation industries, cumulative outbreak incidence was 5.2 times as high in bus and urban transit (129.1) and 3.6 times as high in air transportation (87.7) as in all industries. Annual outbreak incidence in public transportation industries increased by 68.4%, from 11.7 outbreaks per 1,000 establishments in 2020 to 19.7 in 2021, whereas outbreak incidence across all industries increased by 22.9% (from 8.3 to 10.2) during the same period. Numbers of outbreaks increased during COVID-19 surges; the highest monthly number of COVID-19 outbreaks in public transportation industries (79) was reported in December 2021, during the SARS-CoV-2 B.1.1.529 (Omicron) variant surge (Figure).

**FIGURE:**
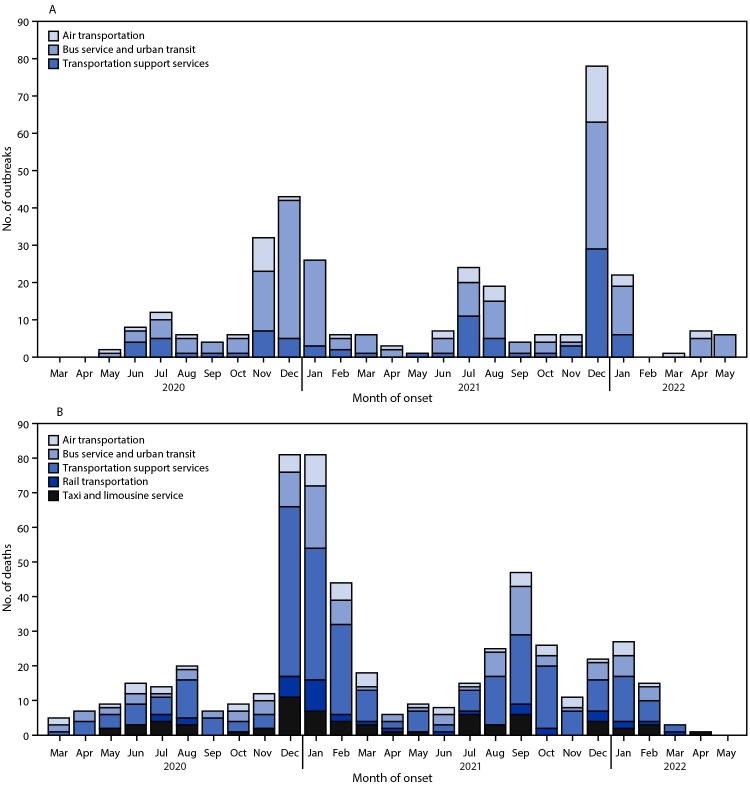
COVID-19 outbreaks* (A) and COVID-19–associated deaths (B) in public transportation industries, by month of onset — California, March 2020–May 2022^†^ * Outbreaks in taxi and limousine services and rail transportation were not included because of low numbers of reported outbreaks. ^†^ No outbreaks or deaths in public transportation industries were reported during January or February 2020.

The cumulative crude mortality rate for all public transportation industries was 174 per 100,000 workers, 1.5 times as high as the rate across all industries (Table 2). Cumulative crude mortality rates among workers in transportation support services (237), rail transportation (242), and bus service and urban transit (211) were approximately twice those across all industries (114). Age-adjusted mortality rates for all public transportation industries combined increased 68.1%, from 55.5 per 100,000 workers during 2020 to 93.3 during 2021 and were 1.5 times as high in 2020 and 1.4 times as high during 2021 as in all industries. The highest monthly number of COVID-19–associated deaths occurred during December 2020 and January 2021 (81 per month); an increase was also observed in September 2021 during California’s SARS-CoV-2 B.1.617.2 (Delta) variant surge (Figure).

## Discussion

COVID-19 outbreak incidence and mortality rates are higher in public transportation industries in California compared with all industries combined. Workers in these industries have continued to report to work throughout the pandemic, and many have jobs involving close, frequent contact with coworkers and the public. Among New York City transit workers who died of COVID-19 early in the pandemic, 57% worked in public-facing positions (*6*). Previous reports in Europe identified elevated mortality risk among public transportation workers; taxi and bus drivers were found to have the highest COVID-19 mortality rates among all occupational groups (*2*,*3*). This report also identified elevated outbreak incidence and mortality rates among bus and urban transit workers, in addition to elevated risk across all public transit industries combined.

Although both outbreak incidence and mortality rates were elevated in the bus and urban transit industry, some differences were observed in other industries. Whereas COVID-19 fatalities were observed in the taxi and limousine industry, no outbreaks were identified, which might reflect the nature of the work (e.g., infrequent direct interaction with other workers) and the challenges of case and outbreak ascertainment with independent contractor work arrangements. Conversely, elevated outbreak incidence in air transportation relative to other transportation industries might partially reflect this industry’s enhanced outbreak identification and contact tracing capability, in addition to other work-related factors, such as duration and intensity of contact with others.

Previous reports of excess all-cause mortality and COVID-19 mortality across occupational groups in California identified elevated mortality rates among non-Hispanic Black and Hispanic workers compared with non-Hispanic White workers in transportation occupations (*1*,*7*). Although examination of outcomes by race and ethnicity was not possible in this analysis because of small numbers and missing data, additional investigation should explore how race, ethnicity, and other socioeconomic factors intersect with occupational risk for COVID-19 in public transportation industries.

The findings in this report are subject to at least six limitations. First, results were limited to California and might not be generalizable to other jurisdictions. Second, a statewide outbreak-reporting mandate was not implemented until January 1, 2021, which might have resulted in underestimation of 2020 outbreaks and limits the ability to compare outbreak incidence between 2020 and 2021. Third, age-adjusted mortality rates could not be calculated for individual industries because of small numbers; other confounding factors (e.g., race and ethnicity, presence of underlying medical conditions, vaccination status, and use of protective measures such as masks) might also affect outbreak and mortality rates and could not be adjusted for in this analysis. Fourth, although the industries analyzed here are public transportation industries, they include some workers who are not in public-facing roles; distinguishing these workers from public-facing workers was not possible in this analysis, and transmission might have occurred between coworkers as well as between workers and members of the public. Fifth, workers could not be distinguished from nonworkers in outbreak-associated case counts and, although death counts were limited to working-age persons and excluded those identified as unemployed or retired, some misclassification of working status remains possible. Decedents were classified by “usual industry,” which might also have led to misclassification of persons with more than one source of employment. Finally, identifying a specific source of COVID-19 exposure for individual patients is challenging because of the occurrence of presymptomatic and asymptomatic transmission and limitations in contact tracing, particularly for workers who come into frequent close contact with many persons; therefore, determining whether and how COVID-19 exposures occurred in the workplace can be difficult, particularly when analyzing aggregate data.

The elevated outbreak incidence identified in public transportation industries suggests higher risk for SARS-CoV-2 workplace exposure among public transportation workers, and elevated mortality rates suggest increased risk for dying from COVID-19. Regardless of whether exposures occur from interactions with the public, coworkers, or other sources, these observations indicate that public transportation workers represent a vulnerable group who should be prioritized for COVID-19 prevention strategies. Such strategies can include targeted vaccination efforts, access to antiviral treatments, public health messaging, and enhanced workplace protection measures, such as improved ventilation and use of well-fitted masks or respirators (e.g., N95s) by workers and members of the public (*8*,*9*).

**Summary**

**What is already known about this topic?**

Workers who perform in-person work and come into close, frequent contact with other workers or the public might be at increased risk for SARS-CoV-2 exposure and infection.

**What is added by this report?**

Public transportation industries in California experienced cumulative COVID-19 outbreak incidence and mortality rates 1.5 times as high as that for all industries; outbreak incidence was 5.2 times as high, and mortality was 1.8 times as high in bus and urban transit industries as in all industries.

**What are the implications for public health practice?**

Public transportation workers should be prioritized for COVID-19 prevention strategies, including vaccination and enhanced workplace protection measures.

## Footnotes

* Laboratory-confirmed cases were defined as those found among persons with detection of SARS-CoV-2 RNA in a clinical or autopsy specimen using a molecular test.

† CDPH recommends that LHDs use Council of State and Territorial Epidemiologists guidelines in making COVID-19 death determinations. https://cdn.ymaws.com/www.cste.org/resource/resmgr/pdfs/pdfs2/20211222_interim-guidance.pdf

§ Corresponding U.S. Census Bureau 2012 industry codes are 6070 (air transportation), 6080 (rail transportation), 6180 (bus service and urban transit), 6190 (taxi and limousine service), and 6290 (services incidental to transportation, referred to here as transportation support services).

¶ The California Employment Development Department defines an establishment as “an economic unit, such as a farm, mine, factory, or store that produces goods or provides services…typically at a single physical location address.” For public transportation industries, this might include a single bus depot at which employees are based, or a single airline office at an airport. If a single establishment reported more than one outbreak during the study period, each outbreak was counted separately (i.e., a single establishment could be responsible for more than one outbreak in the data set). Establishment numbers for 2020 (https://www.labormarketinfo.edd.ca.gov/qcew/cew-select.asp) were extrapolated for 2021–2022 because complete data for 2021–2022 were not available.

** 45 C.F.R. part 46, 21 C.F.R. part 56; 42 U.S.C. Sect. 241(d); 5 U.S.C. Sect. 552a; 44 U.S.C. Sect. 3501 et seq.
